# The Association between* rs2292239* Polymorphism in* ERBB3* Gene and Type 1 Diabetes: A Meta-Analysis

**DOI:** 10.1155/2019/7689642

**Published:** 2019-08-06

**Authors:** Dingjian Wang, Guixia Pan

**Affiliations:** Department of Epidemiology and Biostatistics, School of Public Health, Anhui Medical University, 81 Meishan Road, Hefei, Anhui 230032, China

## Abstract

**Objectives:**

The purpose of this study was to explore the association between* rs2292239 *polymorphism in* ERBB3* gene and type 1 diabetes (T1D).

**Methods:**

A systematic search of studies on the association of* rs2292239* polymorphism in* ERBB3* gene with T1D susceptibility was conducted in PubMed, Web of science, Elsevier Science Direct, and Cochrane Library. Eventually, 9 published studies were included. The strength of association between* rs2292239* polymorphism and T1D susceptibility was assessed by odds ratios (ORs) with its 95% confidence intervals (CIs).

**Results:**

A total of 9 case-control studies, consisting of 5369 T1D patients and 6920 controls, were included in the meta-analysis. This meta-analysis showed significant association between* ERBB3 rs2292239* polymorphism and T1D susceptibility in overall population (A vs. C, OR: 1.292, 95% CI= 1.224-1.364, *P*_H_=0.450, *P*_H_ is P value for the heterogeneity test). Similar results were found in subgroup analysis by ethnicity.

**Conclusions:**

* ERBB3 rs2292239* polymorphism is associated with T1D susceptibility and* rs2292239-*A allele is a risk factor for T1D. However, more large-scale studies are warranted to replicate our findings.

## 1. Introduction

Type 1 diabetes (T1D), a kind of autoimmune disease, is characterized by the progressive loss of insulin-secreting pancreatic beta cells [1–3]. The etiology of T1D is very complex, which is affected by both genetic and environmental factors. Although it may occur at any age, compared with other age groups, the incidence of T1D in children is higher. The incidence of T1D is also significantly different due to region and ethnicity [4, 5]. The destruction of pancreatic beta-cells and the lack of insulin lead to hyperglycemia. T1D patients require exogenous insulin to survival. Therefore, those affected patients are insulin-dependent for life [6, 7]. The complications with T1D are serious.

*ERBB3* gene encodes erb-b2 receptor tyrosine kinase 3 (ErbB3), which is a member of the epidermal growth factor receptor (EGFR) family [8, 9]. ErbB3 interacts more specifically with PIK3 regulatory subunits, which transduce signals downstream to mTOR signaling pathway and in turn regulate insulin production in beta-cells and subsequent glucose metabolism [10].* ERBB3* gene is located on chromosome 12q13;* rs2292239* SNP is located in intron 7 of* ERBB3* gene [11, 12].* ERBB3* gene can also be expressed in pancreatic beta cells.* ERBB3* gene is widely known for its role in cancer, but some studies have shown that the* ERBB3 rs2292239* polymorphism might also play an important role in the pathogenesis of T1D due to immune regulation and apoptosis of beta-cells [13, 14].

Considering the results of* ERBB3* gene polymorphism with T1D susceptibility are inconsistent [15], this discrepancy might be due to studies with small sample size, inadequate statistical power, ethnic differences, and publication bias. Therefore, it is necessary to conduct meta-analysis to explore this association.

## 2. Methods

### 2.1. Search Strategy

A systematic search of studies on the association of* rs2292239* polymorphism in* ERBB3* gene with T1D susceptibility was conducted in PubMed, Web of science, Elsevier Science Direct, and Cochrane Library. Keywords for the search were as follows: (“*ERBB3*” or “*rs2292239*”) and (“Diabetes” or “T1D”) and (“polymorphism” or “variant” or “SNP” or “genotype” or “mutations”). The last search was updated on 15 January, 2019. All relevant studies were retrieved carefully.

### 2.2. Eligibility Criteria

The inclusion criteria were as follows: (I) studies evaluating the association between* rs2292239* polymorphism in* ERBB3* gene and T1D; (II) case-control studies; (III) studies based on humans; (IV) studies providing the detailed relevant genotype data of both case group and control group. Studies were excluded if (I) the study was a review, editorial, abstract, case report, or unpublished article; (II) there were nonhuman studies or animal experiments or cell experiments; (III) studies had no controls or no detailed relevant genotype data.

### 2.3. Data Extraction

The data of the eligible studies were extracted by one investigator (Mr.Wang). The following information was collected: name of first author, year of publication, country, ethnicity, genotyping methods, number of cases and controls in each study, genotype frequency in cases and controls, the value of *P* in Hardy–Weinberg equilibrium (HWE), and other additional information. Different ethnicity descendants were classified as Caucasian and Asian.

### 2.4. Quality Assessment

The qualities of the included studies in this meta-analysis were assessed by another investigator (Dr.Pan). The quality assessment was based on the modified Newcastle-Ottawa Quality Assessment Scale (NOS). The scale consists of eight multiple-choice questions that involve subjects selection, comparability in cases and controls, and assessment of exposure. High-quality response earns a point, totaling up to nine points (the comparability question earns up to two points) [16]. The higher score indicates better quality.

### 2.5. Statistical Analysis

This meta-analysis was conducted following the guidelines from PRISMA and MOOSE statement [17, 18]. Hardy–Weinberg equilibrium (HWE) was evaluated for each study by Chi-square test in control groups. And* P* >0.05 was considered as genetic balance in the study population. All statistical analysis was performed by Stata 12.0 software (Stata Corporation, College Station, TX, USA). OR and 95% CI were used to evaluate the strength of association between* ERBB3 rs2292239* polymorphism and T1D susceptibility. The *χ*^2^ test-based Q statistic was generally used to assess the heterogeneity. Heterogeneity was recognized as statistically significant when *P*_H_ <0.05. According to the value of *P*_H_, we chose fixed-effects model or random-effects model. All subgroups were analyzed. Sensitivity analysis was used to evaluate the influence of individual study on the overall OR. Publication bias was assessed by Funnel's plot, Begg's test, and Egger's test. An asymmetric plot and the *P* value of Egger's test or Begg's test less than 0.05 were considered as significant publication bias.

## 3. Results

### 3.1. Literature Search

**Figure 1:**
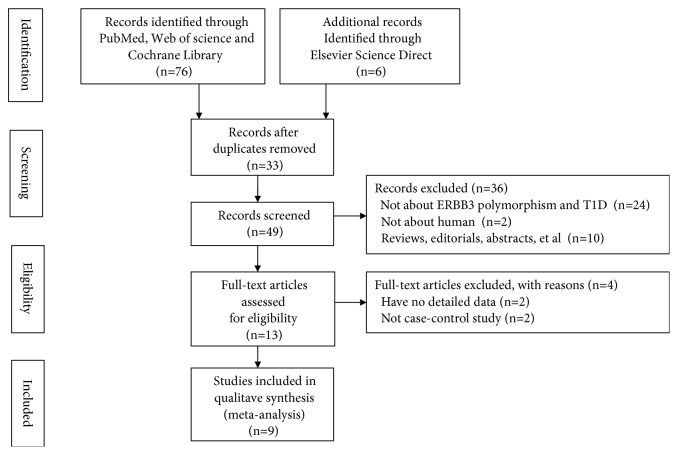
A flowchart of the included and excluded studies.

A total of 82 studies were retrieved from PubMed, Web of science, Elsevier Science Direct, and Cochrane Library. Finally, 9 eligible studies were included in this meta-analysis. A flowchart of the included and excluded studies was shown in Figure 1.

### 3.2. Characteristics of the Included Studies

Table 1 showed the main features of those included studies. Those studies were published from 2009 to 2016. Nine studies involving 5369 T1D patients and 6920 controls were included in this meta-analysis. All included studies met the Hardy–Weinberg equilibrium.

### 3.3. Meta-Analysis of Association between* rs2292239* Polymorphism and T1D Susceptibility

**Figure 2:**
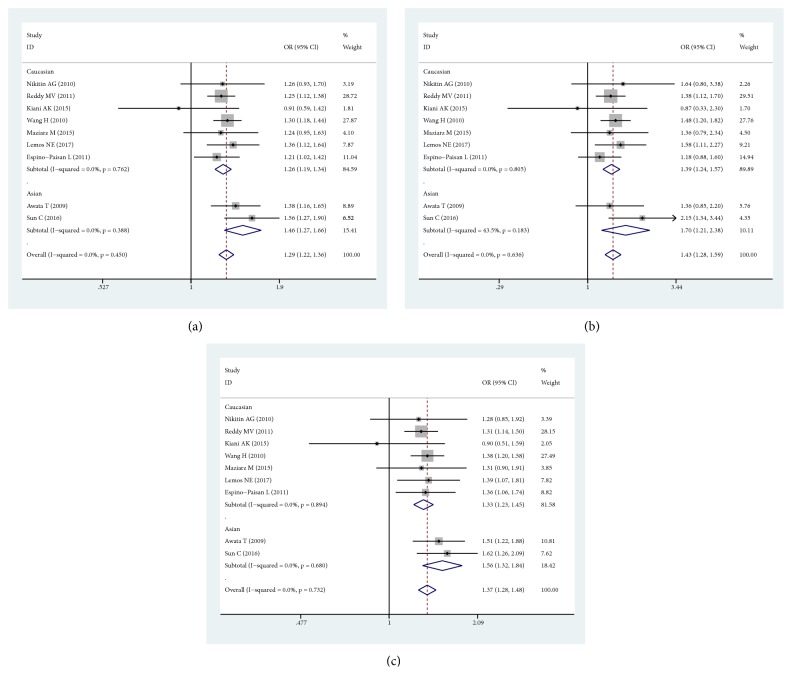
Forest plots for* rs2292239* polymorphism and the risk of T1D. (a) Forest plot for* rs2292239* polymorphism and the risk of T1D under Allelic model. (b) Forest plot for* rs2292239* polymorphism and the risk of T1D under Dominant model. (c) Forest plot for* rs2292239* polymorphism and the risk of T1D under Recessive model.

Meta-analysis indicated significant association between* ERBB3 rs2292239* polymorphism and T1D susceptibility in the overall population (A vs. C, OR: 1.292, 95% CI= 1.224-1.364, *P*_H_=0.450; AA vs. AC+CC, OR: 1.426, 95% CI=1.275-1.594, *P*_H_=0.636; AA+AC vs. CC, OR: 1.374, 95% CI=1.277-1.479, *P*_H_=0.732). Then, subgroup analysis was conducted according to ethnicity. In Caucasians, the significant association was identified in all genetic models (A vs. C, OR: 1.262, 95% CI= 1.190-1.339, *P*_H_=0.762; AA vs. AC+CC, OR: 1.395, 95% CI=1.239-1.570, *P*_H_=0.805; AA+AC vs CC, OR: 1.332, 95% CI=1.228-1.446, *P*_H_=0.894). In Asians, the significant association was also identified in all genetic models (A vs. C, OR: 1.455, 95% CI= 1.273-1.663, *P*_H_=0.388; AA vs. AC+CC, OR: 1.701, 95% CI=1.214-2.384, *P*_H_=0.183; AA+AC vs. CC, OR: 1.560, 95% CI=1.322-1.841, *P*_H_=0.680). The forest plots for* rs2292239* with T1D were shown in Figure 2. The results of subgroup analysis were shown in Table 2.

### 3.4. Heterogeneity and Publication Bias

**Figure 3:**
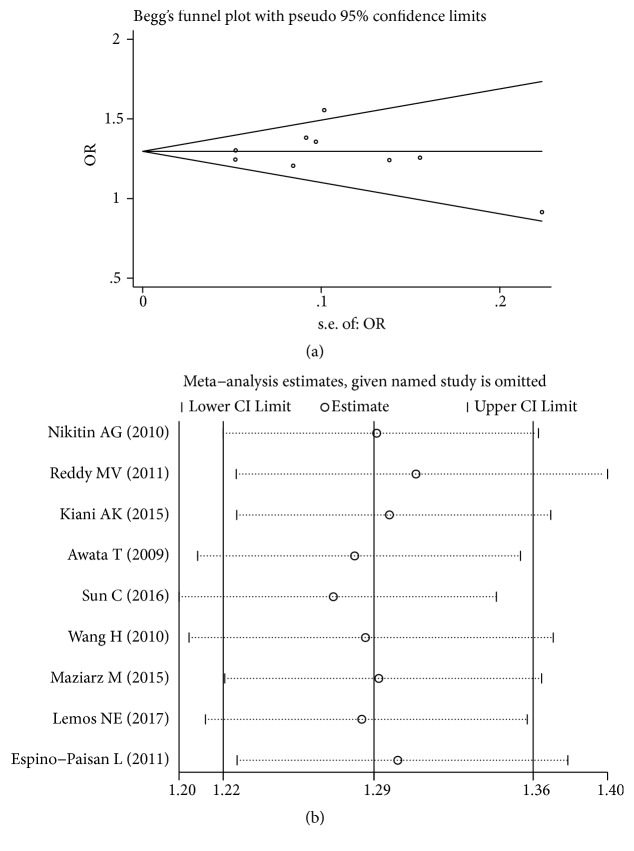
Results of Begg's funnel plot and sensitivity analysis. (a) Begg's funnel plot of* rs2292239*-A with T1D. (b) The sensitivity analysis results of* rs2292239*-A with T1D.

There was no significant heterogeneity across these studies (*P*_H_ >0.05); therefore the fixed-effect models were performed. Publication bias was assessed by Begg's funnel plot, Begg's test, and Egger's test. The Begg's funnel plot showed that the shape of the funnel plot seemed symmetrical (Figure 3(a)). And there was no significant evidence of publication bias found in this meta-analysis (Table 3). The plot of sensitivity analysis was shown in Figure 3(b).

## 4. Discussion

The* ERBB3* gene might play a key role in immune regulation and cytokine-induced pancreatic beta-cell apoptosis, which related to T1D pathogenesis [19–21]. The association between* ERBB3 rs2292239* and T1D susceptibility has been identified in genome-wide association studies, such as TEDDY study [22–24]. However, only few studies evaluated* ERBB3* gene polymorphism and T1D susceptibility in different ethnicities. Therefore, we conducted this meta-analysis. In this meta-analysis, 9 eligible case-control studies including 5369 T1D patients and 6920 controls were analyzed. The present study showed significant association between* ERBB3 rs2292239* polymorphism and T1D susceptibility under all genetic models. Similar results were found in subgroup analysis by ethnicity.

The* ERBB3* gene is located on chromosome 12q13; some studies have shown that the protective genotypes for T1D are associated with higher* ERBB3 *mRNA level in peripheral blood mononuclear cells (PBMCs). ErbB3 protein can be expressed on the surface of CD11c+ cells (dendritic cells and monocytes) in peripheral blood after stimulation. Subjects with protective genotypes have significantly higher percentages of* ERBB3*+ monocytes and dendritic cells, and the percentages of* ERBB3*+ cells are positively correlated with the ability of antigen presenting cells (APCs) to stimulate T cell proliferation [13]. Furthermore, as a member of the epidermal growth factor receptor (EGFR) family, ErbB3 is critical to the activation of the* ERBB3*-PI3K-Akt cascade induced by EGFR/ERBB3 heterodimers. Deficiency or inhibition of the PI3K pathway can lead to reduced numbers of regulatory T cells and autoimmunity [9, 10, 13]. It suggests that the* ERBB3* gene might play an important role in potential T1D pathogenesis.

The* rs2292239* SNP is located in intron 7 of* ERBB3* gene and this SNP has significant functional effects. Some studies have shown that* rs2292239* associates with residual beta-cell function and metabolic,* ERBB3* dysfunction could decrease basal and cytokine-induced apoptosis. The* rs2292239*-A allele might change* ERBB3* expression in immune cells and pancreatic beta-cells, influencing APC function, immunity, and beta-cells apoptosis [9]. Moreover,* rs2292239* has been associated with the production of T1D specific autoantibody [23]. Therefore,* rs2292239* might be involved in T1D pathogenesis and confer disease susceptibility.

Of course, there were some limitations in this meta-analysis. First, the number of included studies was not sufficient, which could not conduct comprehensive analysis. Second, our data of meta-analysis was from retrospective studies, which may be related to the methodological deficiencies because the* rs2292239* polymorphism in* ERBB3* gene can be associated with multitude parameters, such as gender and environment. Third, our meta-analysis did not take those factors into account. All in all, the results should be interpreted with caution.

In conclusion, the present study indicates that* ERBB3 rs2292239* polymorphism is associated with T1D susceptibility. However, it is still necessary to conduct more large-scale studies to further explore the pathogenetic mechanisms of* ERBB3* in T1D.

## Figures and Tables

**Table 1:** The main features of included studies.

Polymorphism	Author	Year	Country	Ethnicity	Case			Control			Genotyping methods	No. of Cases/ Controls	*P* for HWE	NOS score
*rs2292239*					AA (%)	AC (%)	CC (%)	AA (%)	AC (%)	CC (%)				
	Nikitin AG	2010	Russia	Caucasian	19 (10.73)	85 (48.02)	73 (41.24)	14 (6.83)	94 (45.85)	97 (47.32)	PCR-RFLP	177/205	0.165	5
	Reddy MV	2011	USA	Caucasian	207 (14.44)	676 (47.14)	551 (38.42)	203 (10.89)	824 (44.21)	837 (44.90)	TaqMan	1434/1864	0.993	7
	Kiani AK	2015	Pakistan	Caucasian	8 (8.79)	37 (40.66)	46 (50.55)	10 (10.00)	42 (42.00)	48 (48.00)	TaqMan	91/100	0.855	7
	Awata T	2009	Japan	Asian	46 (6.26)	299 (40.68)	390 (53.06)	29 (4.67)	200 (32.21)	392 (63.12)	TaqMan	735/621	0.591	6
	Sun C	2016	China	Asian	38 (10.44)	152 (41.76)	174 (47.80)	37 (5.15)	252 (35.05)	430 (59.81)	NA	364/719	0.992	5
	Wang H	2010	USA	Caucasian	207 (14.44)	676 (47.14)	551 (38.42)	191 (10.24)	812 (43.54)	862 (46.22)	TaqMan	1434/1865	0.991	6
	Maziarz M	2015	Sweden	Caucasian	44 (15.77)	134 (48.03)	101 (36.20)	23 (12.11)	86 (45.26)	81 (42.63)	PCR-RFLP	279/190	0.981	6
	Lemos NE	2017	Brazil	Caucasian	78 (18.53)	178 (42.28)	165 (39.19)	64 (12.55)	205 (40.20)	241 (47.25)	TaqMan	421/510	0.052	7
	Espino Paisan L	2011	Spain	Caucasian	82 (18.89)	225 (51.84)	127 (29.26)	139 (16.43)	403 (47.64)	304 (35.93)	TaqMan	434/846	0.780	6

HWE: Hardy–Weinberg equilibrium; PCR-RFLP: polymerase chain-reaction fragment length polymorphism; NOS: Newcastle-Ottawa Quality Assessment Scale.

**Table 2:** The results of meta-analysis.

Polymorphism	Allelic model		Dominant model		Recessive model	
*rs2292239*	A versus C		AA versus AC+CC		AA+AC versus CC	
	OR (95% CI)	*P*_H_	OR (95% CI)	*P*_H_	OR (95% CI)	*P*_H_
overall	1.292(1.224-1.364)	0.450	1.426(1.275-1.594)	0.636	1.374(1.277-1.479)	0.732
Ethnicity						
Caucasian	1.262(1.190-1.339)	0.762	1.395(1.239-1.570)	0.805	1.332(1.228-1.446)	0.894
Asian	1.455(1.273-1.663)	0.388	1.701(1.214-2.384)	0.183	1.560(1.322-1.841)	0.680

*P*_H_: the value of *P* in the heterogeneity test.

**Table 3:** The results of publication bias.

Polymorphism	Test of publication bias			
*rs2292239*	Begg's test		Egger's test	
	*Z*	*P*	*t*	*P*
Allelic model	-0.10	1.000	-0.12	0.909
Dominant model	0.10	0.917	0.31	0.769
Recessive model	1.15	0.251	-0.40	0.698
